# Rapid Phenotypic Detection of Microbial Resistance in Gram-Positive Bacteria by a Real-Time Laser Scattering Method^†^

**DOI:** 10.3389/fmicb.2017.01064

**Published:** 2017-06-14

**Authors:** Evgeny A. Idelevich, Matthias Hoy, Dennis Görlich, Dennis Knaack, Barbara Grünastel, Georg Peters, Matthias Borowski, Karsten Becker

**Affiliations:** ^1^Institute of Medical Microbiology, University Hospital MünsterMünster, Germany; ^2^Institute of Biostatistics and Clinical Research, University of MünsterMünster, Germany

**Keywords:** rapid diagnostics, resistance detection, susceptibility, laser scattering, BacterioScan

## Abstract

We developed a methodology for antimicrobial susceptibility testing (AST) based on the BacterioScan^TM^216R laser scattering technology, using methicillin resistance in *Staphylococcus aureus* and vancomycin resistance in enterococci as exemplar for important resistance phenotypes. Fifty methicillin-resistant (MRSA) and 50 methicillin-susceptible (MSSA) *S. aureus*, as well as 50 vancomycin-resistant enterococci (VRE) and 50 vancomycin-susceptible enterococci (VSE) isolates were used for the study. Optimal test conditions were derived by investigating the effects of inoculum size, medium, incubation temperature and broth filtration. We proposed four different statistical approaches for rapid discrimination between resistant and susceptible bacteria. The statistical approach based on raw measurements of bacterial concentrations delivered sensitivity of 100% and specificity of 94% for discrimination between MRSA and MSSA already after 3 hours of incubation. Categorical agreement of ≥90% was achieved after 140 min with this approach. Differentiation between VRE and VSE was possible with 98% sensitivity and 92% specificity after 3 hours, using a sophisticated statistical approach based on concentration slopes derived from the raw concentration measurements. This approach provided categorical agreement of ≥90% after 165 min. The sensitivity and specificity estimates were confirmed by leave-one-out cross validation. In conclusion, the phenotypic AST methods developed in this study are promising for rapid detection of MRSA and VRE. The development and application of this technology would allow early detection of the resistant pathogens, thus facilitating swift change to the targeted antimicrobial treatment as well as timely initiation of appropriate infection control measures. Further studies are warranted to validate this approach for the detection of other resistance phenotypes, including direct testing from clinical specimens.

## Introduction

Increasing prevalence of multidrug resistant microorganisms (MDROs) emphasizes the need for their rapid detection and identification (Roca et al., 2015). Early knowledge of pathogen’s species and antimicrobial susceptibility profile allows prompt change from empirically chosen antimicrobial treatment to the appropriate and targeted therapy (Doern et al., 1994; Idelevich et al., 2015). Administration of specific antibiotics not only enables effective treatment but also allows to minimize collateral damage on physiological flora and to prevent the development of resistant bacteria (Goldstein, 2011). Furthermore, rapid detection of MDROs facilitates swift initiation of infection control measures (Roca et al., 2015). While virtually immediate identification of microbial cultures has become available after the introduction of matrix-assisted laser desorption ionization time-of-flight mass spectrometry (MALDI-TOF MS) in clinical microbiology (Bizzini and Greub, 2010), antimicrobial susceptibility testing (AST) still takes long time (van Belkum et al., 2013). In diagnostic routine, the results of AST are usually available only on the next day or later after test initiation even though optimized protocols may accelerate AST (Kerremans et al., 2008; Idelevich et al., 2014a,b). Molecular methods can provide information on the presence of resistance genes more rapidly, but they are expensive and do not always correspond with the phenotypic testing (Jorgensen and Ferraro, 2009). Thus, rapid methods for detection of resistant phenotypes are urgently needed.

In this study, we focused on methicillin resistance in *S. aureus* and vancomycin resistance in enterococci as exemplar for resistance phenotypes with enormous medical and socio-economic burden (Köck et al., 2010). Methicillin-resistant *Staphylococcus aureus* (MRSA) is a major nosocomial pathogen and its rapid differentiation from methicillin-susceptible *S. aureus* (MSSA) isolates is crucial for the initiation of adequate infection control measures and antimicrobial therapy (Köck et al., 2014). Vancomycin-resistant enterococci (VRE) represent another multidrug resistant organism with increasing prevalence and similar challenges for the health care system (Gastmeier et al., 2014; Humphreys, 2014).

Here, we aimed at developing methods for AST that are based on the laser scattering technology implemented in the BacterioScan^TM^216R (BacterioScan Inc., St Louis, MO, United States) instrument. BacterioScan uses the laser scattering technology to quantify bacteria in up to 16 liquid samples in real time. The combination of the laser scattering technology with sophisticated statistical real-time analyses may provide a promising approach for fast and reliable discrimination between susceptible and resistant strains. We propose four statistical approaches for this purpose. The study’s main objectives were (i) to quantify the discriminatory performance of each method in terms of sensitivity, specificity, and categorical agreement and (ii) to determine the incubation time required for a reliable discrimination between resistant and susceptible isolates.

## Materials and Methods

### Investigation of Optimal Test Conditions

Comparative investigation of optimal conditions for AST by laser scattering included the evaluation of the effects of inoculum size, various media, broth filtration as well as the effect of incubation temperature. All experiments were performed in triplicate.

#### Effect of Inoculum Size

After preparation of bacterial suspensions of reference strains *S. aureus* ATCC BAA-44 and *Escherichia coli* ATCC 25922 in brain-heart infusion (BHI) broth (Merck, Darmstadt, Germany) with 0.5 McFarland turbidity using a nephelometer (Densimat, bioMérieux, Marcy l’Etoile, France), serial dilutions in BHI were made to produce suspensions with estimated 10-fold concentrations from 1 cfu/ml to 1 × 10^8^ cfu/ml. Additionally, a suspension with 5 × 10^5^ cfu/ml was prepared, which reflects the bacterial density recommended as starting inoculum for AST by the International Organization for Standardization ISO (2006) and the Clinical Laboratory Standards Institute CLSI (2015) guidelines. The real cell concentration in these suspensions was confirmed by vital cell counting after plating of a sample onto tryptic soy agar (TSA) plates in triplicate. Two ml samples of each dilution and a sterile control sample were added to the cuvettes of the BacterioScan instrument, and the bacterial concentration was measured for 24 h at 35°C. Applying different starting inocula, the time until fivefold and 10-fold increase in inoculum was documented to determine the length of time needed to detect the growth by laser scattering.

#### Effect of Medium

Bacterial suspensions with 0.5 McFarland turbidity (Densimat, bioMérieux) were prepared in cation-adjusted Mueller-Hinton broth (CA-MHB, BD Diagnostics, Heidelberg, Germany), BHI broth (Merck), tryptic soy broth (TSB, BD Diagnostics, Germany) and lysogeny broth (LB, BD Diagnostics, Germany) for *S. aureus* ATCC BAA-44 and *Escherichia coli* ATCC 25922. All broths were used unfiltered. The suspensions were diluted 1:200 to produce an inoculum concentration of approximately 5 × 10^5^ cfu/ml. The real cell concentration was verified by vital cell count after plating onto TSA plates. Two ml samples were measured in BacterioScan for 24 h at 35°C. The time until fivefold and 10-fold concentration increase was recorded for comparison of the length of times needed to detect the growth using different media.

#### Effect of Broth Filtration

Suspensions of 0.5 McFarland turbidity (Densimat, bioMérieux) were prepared in CA-MHB and BHI broth for *S. aureus* ATCC BAA-44 and *Escherichia coli* ATCC 25922. One sample set was filtered through a 0.2 μl syringe filter (Sarstedt, Nümbrecht, Germany) while other sample set was left unfiltered. The suspensions were diluted 1:200 to produce an inoculum concentration of approximately 5 × 10^5^ cfu/ml (confirmed by vital cell count). Two ml samples were incubated in BacterioScan for 8 h at 35°C. The time until fivefold and 10-fold increase in inoculum was documented to compare the length of time needed for growth detection using filtered or unfiltered broth.

#### Effect of Incubation Temperature

Applying the BacterioScan instrument, the effect of incubation temperature on staphylococcal AST was investigated on MSSA reference strains ATCC 29213 and ATCC 25923 as well as MRSA reference strains ATCC BAA-44 and ATCC 43300 using CA-MHB as medium. One ml of cefoxitin solution was added to 1 ml of bacterial suspension to produce a final inoculum of 5 × 10^5^ cfu/ml and a final cefoxitin concentration of 4 μg/ml (breakpoint concentration of cefoxitin, which allows the differentiation between MRSA and MSSA, according to the European Committee on Antimicrobial Susceptibility Testing EUCAST (2016) and CLSI (2016). The temperatures investigated were 30°C, 33°C, 34°C, 35°C, 36°C, and 37°C (Supplementary Figure S1). Real microbial concentrations were confirmed by vital cell count. Controls without antibiotics were implemented.

### Bacterial Strains

Fifty MRSA and 50 MSSA isolates, as well as 50 vancomycin-resistant *E. faecium* (VR-*E. faecium*) and 50 vancomycin-susceptible *E. faecium* (VS-*E. faecium*) clinical isolates were used in the study. Only one isolate per patient was included. Species identification was confirmed by MALDI-TOF MS. Methicillin resistance in *S. aureus* and vancomycin resistance in *E. faecium* isolates were confirmed by GenoType MRSA (Hain Lifescience, Nehren, Germany) assay and *vanA/vanB* PCR, respectively. Among VREs, 24 and 26 isolates were *vanA*-positive and *vanB*-positive, respectively.

### Antimicrobial Susceptibility Testing

#### Reference Method

Minimum inhibitory concentrations (MIC) of cefoxitin for *S. aureus* and vancomycin for *E. faecium* were determined by broth microdilution (BMD) reference method according to the ISO 20776-1 (2006) and CLSI (2015) guidelines. Cefoxitin and vancomycin powders were purchased from Sigma-Aldrich (Saint Louis, MO, United States). The range of tested concentrations was 0.25–128 μg/ml for both antimicrobials. The tests were performed in triplicate on different days and median MIC values were used for analysis. Reference strains *S. aureus* ATCC 29213 and *E. faecalis* ATCC 29212 were used on every testing day for staphylococci and enterococci, respectively.

#### Measuring Microbial Concentration by Forward Laser Scattering

Quantitative estimation of the microbial concentration was performed using the BacterioScan^TM^216R instrument, which uses narrow angle forward laser scattering as a sensitive optical method for measuring light scattered by bacteria suspended in a liquid sample. Multiple simultaneous measurements of the forward scattering and optical density performed over time, in combination with proprietary calculation algorithms, provide accurate estimation of microbial concentrations even at low cfu/ml levels (Anbazhagan and Regelman, 2015). The tabletop instrument accommodates up to four disposable multi-cuvettes, each of them containing four wells of approximately 2 ml volume. Additionally to the possibility of incubation at room temperature, onboard incubation allows temperatures between 30°C und 42°C.

**FIGURE 1:**
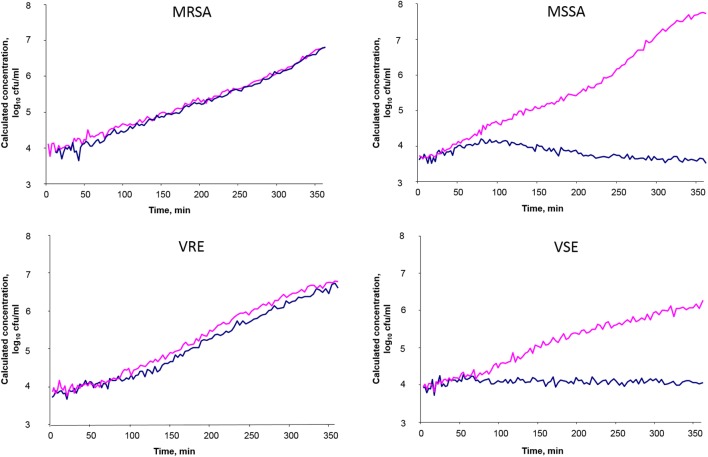
Examples of phenotypic detection of microbial resistance in clinical *Staphylococcus aureus* and *Enterococcus faecium* isolates using real-time laser-scattering method. The samples with antibiotic are indicated in blue, and the growth control samples without antibiotic are indicated in red. Cefoxitin was used in breakpoint concentration 4 μg/ml for detection of methicillin resistance in *S. aureus*; vancomycin was used in breakpoint concentration 4 μg/ml for detection of vancomycin resistance in enterococci.

0.5 McFarland turbidity (Densimat) suspensions were prepared in CA-MHB (BD Diagnostics) and diluted to produce a starting inoculum concentration of approximately 5 × 10^5^ cfu/ml after adding the antibiotic. The cell concentration in these suspensions was confirmed by vital cell count after plating onto TSA plates in triplicate. The samples were incubated in the BacterioScan^TM^216R device for 6 h. For detection of methicillin resistance in staphylococci, cefoxitin was used in the breakpoint concentration of 4 μg/ml as recommended by CLSI (2016) and EUCAST (2016) and the samples were incubated at 34°C. For detection of vancomycin resistance in enterococci, the vancomycin was added to produce the final breakpoint concentration 4 μg/ml (CLSI, 2016; EUCAST, 2016), followed by the incubation of samples at 35°C. Antibiotic-free growth controls were included for each isolate. The measurements were taken automatically approximately every 3 min for each sample, and then converted into sequences of one measurement per minute by simple linear interpolation. Example growth curves are given in **Figure 1**. While the growth of susceptible strains is inhibited by antibiotic, the growth curves with a given antibiotic compound are comparable to those of the growth control without antibiotic in resistant strains.

### Statistical Analysis

#### Optimal Test Conditions

The effects of inoculum size, various media, and broth filtration were analyzed by univariate ANOVA with Tukey’s test using IBM SPSS Statistics 23 software (IBM, Armonk, NY, United States).

#### Approaches to Discriminate between Resistant and Susceptible Bacteria

Four different approaches to discriminate between resistant and susceptible bacteria based on data delivered by the laser scattering methodology were investigated (Supplementary Figure S2) based on

(a) concentration,
(b) ratio of concentrations (sample with antibiotics vs. growth control),
(c) concentration slope, and
(d) ratio of concentration slopes (sample with antibiotics vs. growth control).

In approach (a) the concentration measurements *x*_t_ at time *t*, say *t* = 120 [minutes], of the 50 resistant and 50 susceptible isolates (each with added antibiotic) were used to perform a receiver-operator-curve (ROC) analysis, in order to determine the discrimination cutoff at time *t* = 120 that delivered the highest sum of sensitivity and specificity. This was done for all times *t* = 60,…,360. Approach (b) was equal to (a), except that the concentration ratio *x*_t_/*y*_t_ instead of the concentration *x*_t_ was considered for ROC analyses, where *y*_t_ denotes the concentration of the growth control that corresponds to *x*_t_ In approach (c), the slope of bacterial growth *s*_t_ was derived from the time series of concentration measurements *x*_t_ at time *t* = 60,…,360 by means of the SCARM filter (Borowski and Fried, 2014). The ROC analyses were then performed on *s*_t_ at time *t* = 60,…,360. The SCARM is a real-time filter, i.e., it provides a slope estimate *s*_t_ as soon as the concentration *x*_t_ has been measured. Approach (c) could therefore be applied in practice. The rationale for choosing this approach as well as more details about the SCARM estimation of *s*_t_ are given in the Supplementary Description. In approach (d), the ratio *s*_t_/*u*_t_ was considered for ROC analysis, where *u*_t_ denotes the SCARM slope estimate of the growth control *Y*_t_ that corresponds to *x*_t_

For each approach (a)-(d), the ROC analyses delivered a best discrimination cutoff and its sensitivity and specificity estimates (with 95% confidence intervals) at time *t* = 60,…,360. Additionally, the incubation duration until overall categorical agreement (CA) reached ≥90%, which is an acceptance limit for accuracy of susceptibility testing recommended by ISO (2007) 20776-2 guideline, was calculated. Since cutoff determination and estimation of sensitivity and specificity were carried out on the same data, probably leading to biased estimates, we calculated all sensitivity and specificity estimates and their 95% confidence intervals by leave-one-out cross-validation, too. All analyses described here were performed using R, version 3.3.2.

## Results

### Investigation of Optimal Test Conditions

For both reference strains, no significant difference between starting inocula from 5 × 10^5^ to 1 × 10^8^ was demonstrated regarding time to detection of fivefold or 10-fold increase in bacterial concentration. However, this time was significantly longer for lower starting inocula (Supplementary Table S1). Neither the four liquid media nor the broth filtration influenced the time to growth detection significantly (Supplementary Tables S2, S3). The MRSA reference strain ATCC BAA-44 grew, as expected, in the presence of cefoxitin at incubation temperatures 30°C, 33°C, 34°C, 35°C, 36°C. However, at 37°C it was completely inhibited by cefoxitin, thus behaving like an MSSA (Supplementary Figure S1). The growth of the other MRSA reference strain ATCC 43300 was also delayed to some extent at 37°C, but the growth was restored after approximately 12 hours (data not shown). The MSSA reference strains ATCC 29213 and ATCC 25923 were inhibited by cefoxitin at all incubation temperatures, as expected (data not shown).

### Standard Resistance Detection

MIC_50_, MIC_90_ and MIC range, as determined by broth microdilution, were 4 μg/ml, 4 μg/ml, 2–4 μg/ml, and 64 μg/ml, ≥256 μg/ml, 16–≥256 μg/ml for MSSA and MRSA, respectively. For VS-*E. faecium* and VR-*E. faecium*, MIC_50_, MIC_90_ and MIC ranges were 1 μg/ml, 1 μg/ml, 0.5 – 2 μg/ml and ≥256 μg/ml, ≥256 μg/ml, 32–≥256 μg/ml, respectively.

### Performance of the Approaches to Discriminate between Resistant and Susceptible Bacteria

Estimates of the sensitivity and specificity of the approaches (a)–(d) applied to discriminate between MRSA and MSSA are presented in **Table 1**. For all approaches, both the sensitivity and specificity were >90% after 4 h, and >98% after 5 h. Interestingly, the approaches (a) and (c), i.e., those approaches that do not require growth controls, performed better than approaches (b) and (d). For approach (a) and (c), the estimates of sensitivity and specificity were >90% already after 3 h, with lower bounds of the 95% CIs of >89.4% for sensitivity and >83.5% for specificity. The overall categorical agreement of ≥90% was achieved after 140 and 147 min with approaches (a) and (c), respectively, followed by 151 and 166 min with approaches (b) and (d), respectively.

**Table 1:** Accuracy of the approaches **(a)**–**(d)** to discriminate between methicillin-resistant and methicillin-susceptible *Staphylococcus aureus.*

Approach	(a)				(b)				(c)				(d)			
				
Min.	SE (%)	(95% CI)	SP (%)	(95% CI)	SE (%)	(95% CI)	SP (%)	(95% CI)	SE (%)	(95% CI)	SP (%)	(95% CI)	SE (%)	(95% CI)	SP (%)	(95% CI)
60	84	(70.9–92.8)	48	(33.7–62.6)	52	(37.4–66.3)	78	(64–88.5)	66	(51.2–78.8)	64	(49.2–77.1)	48	(33.7–62.6)	76	(61.8–86.9)
120	82	(68.6–91.4)	80	(66.3–90)	80	(66.3–90)	88	(75.7–95.5)	78	(64–88.5)	84	(70.9–92.8)	68	(53.3–80.5)	90	(78.2–96.7)
180	100	(92.9–100)	94	(83.5–98.7)	86	(73.3–94.2)	98	(89.4–99.9)	98	(89.4–99.9)	96	(86.3–99.5)	84	(70.9–92.8)	100	(92.9–100)
240	100	(92.9–100)	100	(92.9–100)	94	(83.5–98.7)	94	(83.5–98.7)	100	(92.9–100)	100	(92.9–100)	100	(92.9–100)	100	(92.9–100)
300	100	(92.9–100)	100	(92.9–100)	100	(92.9–100)	100	(92.9–100)	100	(92.9–100)	100	(92.9–100)	100	(92.9–100)	100	(92.9–100)
360	100	(91.6–100)	98	(89.4–99.9)	100	(91.6–100)	98	(89.4–99.9)	100	(91.6–100)	98	(89.4–99.9)	100	(91.6–100)	100	(92.9–100)


*SE, Sensitivity; SP, Specificity; (a) Discrimination based on concentrations, (b) Discrimination based on ratios of concentrations (sample with antibiotics vs. growth control), (c) Discrimination based on concentration slopes, (d) Discrimination based on ratios of concentration slopes (sample with antibiotics vs. growth control).*

**Table 2** shows the sensitivity and specificity estimates for VR-*E. faecium* and VS-*E. faecium*. The approaches (c) and (d), i.e., the approaches based on concentration slopes, performed best, with slightly larger values for approach (c). This approach delivered sensitivity and specificity of >90% after 3 h, where the lower bounds of the 95% CIs were >89.4% for sensitivity and >80.8 for specificity. While approaches (c) and (d) provided categorical agreement ≥90% after 165 min and 164 min, respectively, this limit was not achieved with approach (a) before 215 min, and was not achieved at all with approach (b).

**Table 2:** Accuracy of the approaches **(a)**–**(d)** to discriminate between vancomycin-resistant and vancomycin-susceptible *Enterococcus faecium.*

Approach	(a)				(b)				(c)				(d)			
				
Min.	SE (%)	(95% CI)	SP (%)	(95% CI)	SE (%)	(95% CI)	SP (%)	(95% CI)	SE (%)	(95% CI)	SP (%)	(95% CI)	SE (%)	(95% CI)	SP (%)	(95% CI)
60	90	(78.2–96.7)	30	(17.9–44.6)	90	(78.2–96.7)	44	(30–58.7)	56	(41.3–70)	62	(47.2–75.3)	48	(33.7–62.6)	78	(64–88.5)
120	82	(68.6–91.4)	66	(51.2–78.8)	78	(64–88.5)	84	(70.9–92.8)	86	(73.3–94.2)	70	(55.4–82.1)	80	(66.3–90)	82	(68.6–91.4)
180	92	(80.8–97.8)	86	(73.3–94.2)	90	(78.2–96.7)	82	(68.6–91.4)	98	(89.4–99.9)	92	(80.8–97.8)	94	(83.5–98.7)	98	(89.4–99.9)
240	92	(80.8–97.8)	90	(78.2–96.7)	92	(80.8–97.8)	80	(66.3–90)	98	(89.4–99.9)	92	(80.8–97.8)	98	(89.4–99.9)	94	(83.5–98.7)
300	90	(78.2–96.7)	98	(89.4–99.9)	96	(86.3–99.5)	76	(61.8–86.9)	100	(92.9–100)	98	(89.4–99.9)	100	(92.9–100)	92	(80.8–97.8)
360	90	(78.2–96.7)	100	(92.9–100)	96	(86.3-99.5)	78	(64–88.5)	98	(89.4–99.9)	96	(86.3–99.5)	98	(89.4–99.9)	86	(73.3–94.2)


*SE, Sensitivity; SP, Specificity; (a) Discrimination based on concentrations, (b) Discrimination based on ratios of concentrations (sample with antibiotics vs. growth control), (c) Discrimination based on concentration slopes, (d) Discrimination based on ratios of concentration slopes (sample with antibiotics vs. growth control).*

The sensitivity and specificity estimates and their 95% CIs that were obtained by leave-one-out cross-validation (Supplementary Tables S4, S5) were very close to those presented in **Tables 1**, **2**, indicating their reliability.

## Discussion

Rapid AST is a function of several components addressed in our study: optimal testing conditions for rapid and correct exhibiting of phenotypic resistance, susceptible growth detection technology, and sophisticated statistical algorithms for early differentiation between resistant and susceptible phenotypes.

Our investigations of the effects of different testing conditions for the optimal, i.e., rapid and exact, AST revealed that it was unnecessary to change conditions which are recommended by ISO (2006) and CLSI (2015) guidelines for reference broth microdilution method. Use of other broths than recommended CA-MHB did not accelerate the detection of growth (Supplementary Table S2). Similarly, inoculum sizes higher than recommended 5 × 10^5^ cfu/ml did not considerably accelerate the detection of growth. Merely, the inocula lower than 5 × 10^5^ cfu/ml resulted in the significantly delayed growth detection, as expected, because they first need to reach the instrument’s lower detection limit during the incubation (Supplementary Table S1). Broth filtration might be deemed helpful for testing by optical systems, but it also did not influence the time to growth detection considerably.

Thus, we demonstrated that there is generally no reason to alter the BacterioScan testing conditions compared to those recommended for the reference method. On the other hand, it should be kept in mind that the introduction of deviations in test conditions might affect the test results. This can be best exemplified by the effect of different incubation temperatures. Elevation of incubation temperature up to only 37°C had a tremendous effect on the results of staphylococcal AST in our experiments. The MRSA strain ATCC BAA-44, which grew as expected in the presence of breakpoint cefoxitin concentration at temperatures from 30 to 36°C, was inhibited with the same antibiotic concentration at 37°C, i.e., it behaved as MSSA at higher temperature (Supplementary Figure S1). This effect was observed in MRSA strain ATCC BAA-44, but not in MRSA strain 43300, demonstrating the strain-dependence of this effect. The loss of methicillin resistance in some *S. aureus* strains at higher temperature is known and was described early by other investigators (Asheshov, 1966). Interestingly, such findings provide experimental background for the hypothesis that β-lactam antibiotics can be used in treatment of infections caused by MRSA, e.g., in the framework of combination regimens with non-β-lactam antibiotics. Clinical studies investigating the practical benefit of such regimens are warranted (Dhand et al., 2011; Poulsen et al., 2013).

Another example of unfavorable effects of deviating test conditions from the standard is the so-called inoculum effect well described for some groups of antimicrobials, particularly for β-lactam antibiotics (Sabath et al., 1975; Brook, 1989). Therefore, unnecessary deviations in test conditions should be strictly avoided. As a result of the preliminary experiments and the theoretical considerations, we decided to keep the test conditions for the main experiments (broth type, inoculum size, antibiotic concentration) as close as possible to the official recommendations for AST (ISO, 2006; CLSI, 2016; EUCAST, 2016). Only the sample volume (2 ml) had to be adapted to the requirements of the test device.

The key difference and concurrent major advantage of the approaches investigated in this study was the earlier evaluation of growth as compared to the standard testing methods, i.e., the visual reading of turbidity after 16–20 h with the reference broth microdilution method (ISO, 2006; CLSI, 2016). This was accomplished using the technology of cell counting implemented in the BacterioScan instrument in combination with the statistical algorithms for early differentiation between multiplying or inhibited microorganisms.

Reliable differentiation between MSSA and MRSA and between VSE and VRE was possible already after less than 3 h (**Tables 1**, **2**). Results from leave-one-out cross-validation were similar, indicating that the proposed methods would be as accurate and fast when applied to further concentration measurements of staphylococci and enterococci.

Attempts were made to find out the optimal method of statistical analysis, which allows for discriminating between susceptible and resistant isolates as early as possible. Interestingly, incorporating the growth control data into the statistical approaches [approaches (b) and (d)] resulted in neither faster nor more accurate differentiation between susceptible and resistant isolates of both staphylococci and enterococci. It even reduced accuracy in some situations, which might be due to the additional variability coming from each isolate’s growth control. While testing staphylococci, the slope estimating approaches (c) and (d) had no advantage over the simpler approaches (a) and (b) that are based on concentrations only. However, the slope estimating approaches provided more accurate and faster discrimination between VRE and VSE. This is most probably because the concentrations of MRSA and MSSA differed considerably after 3 to 4 hours, whereas the concentrations of VRE and VSE did not (Supplementary Description). However, the *slopes* of VRE and VSE did differ considerably quite early, therefore making the slope estimating approaches (c) and (d) superior to the simpler “concentration approaches” (a) and (b) for enterococci. Since such structures are expectable also in concentration time series of other bacteria, we think that the slope estimating approaches can be more promising for some microorganism groups. Moreover, we are convinced that refining and combining statistical approaches could improve growth-based AST even further.

Early studies have identified light scattering technology as a promising tool for detecting microbial resistance within a short time (Berkman et al., 1970; Wyatt, 1972; Murray et al., 1980). Already at that time, efforts were made to develop instruments for rapid AST (Phillips, 1971; Stull, 1973). BacterioScan device has been recently introduced as an instrument for microbial quantification using laser scattering (Anbazhagan and Regelman, 2015). The device has an advantage of compact design and easy-to-use cuvettes, which make it promising particularly for the on-demand single resistance tests. Two very recent studies have investigated laser scattering technology using BacterioScan instrument for AST (Bugrysheva et al., 2016; Hayden et al., 2016). Bugrysheva et al. have demonstrated that laser scattering technology reduced the time to AST result by 50 to 75% for biothreat bacteria *Bacillus anthracis*, *Yersinia pestis*, and *Burkholderia pseudomallei*, as compared to conventional methods (Bugrysheva et al., 2016). Bugrysheva et al. (2016) discriminate between growing resistant and susceptible bacteria by comparing CFU/ml counts for a bacterial strain with and without an antimicrobial within the 30-min intervals by means of *p*-values from paired *t*-tests and Wilcoxon-tests. Hayden et al. (2016) have shown accurate determination of antimicrobial susceptibility using three isolates each of *S. aureus*, *E. coli*, and *Pseudomonas aeruginosa*. Hayden et al. (2016) consider rules based on areas under the curve of laser scattering optical density measurements to determine antimicrobial susceptibility. As a proof-of-principle, the authors demonstrate a high degree of categorical concordance with two commercial methods for AST (Hayden et al., 2016). However, the quantification of reliability to discriminate between resistant and susceptible bacteria with the approaches used in these both studies is limited due to a low number of isolates used.

In our study, we directly used the bacterial concentrations calculated by the instrument for the analysis. We did not calibrate the calculated concentrations to the bacterial concentrations determined by the plating of suspensions for colony count, as it was done in another study (Bugrysheva et al., 2016). The statistical analysis was based on the concentration data directly generated by the instrument (calculated cfu/ml). The comparability of these primary data between resistant and susceptible isolates as well as samples with and without antibiotics is obvious because the proprietary software generates all data sets in the same way. In contrast, transforming those calculated concentrations to “calibrated cfu/ml” has no additional benefit for analysis and, to our opinion, can even result in additional bias. This is because the real concentrations determined by colony count method for a chosen “calibrating strain” does not necessarily reflect the concentrations of other isolates even within the same species. Furthermore, growth in chains, as pointed out by Bugrysheva et al. (2016), or in clusters of cohering cells may result in colony counting error. This could also apply to enterococci and staphylococci used in our study.

Since this study investigated the detection of single resistance phenotypes by using only one antibiotic against particular pathogen, the information on susceptibility toward other antibiotics is missing. Simultaneous testing of multiple antimicrobials would provide information on alternative treatment options and, thus, facilitate prompt and correct treatment choice.

## Conclusion

The phenotypic AST methods developed in this study are promising for rapid detection of MRSA and VRE. Further studies are required to investigate and validate these approaches for detecting other important resistance phenotypes. Future studies should also focus on direct testing from clinical specimens.

## Author Contributions

EI and KB conceived and designed the experiments; MH, BG, and EI performed the experiments; EI, MH, DG, DK, GP, MB, and KB analyzed the data; MB and DG developed statistical algorithms and performed statistical analyses; EI wrote the manuscript, with input from MH, DG, DK, GP, MB, and KB.

## Conflict of Interest Statement

EI received congress travel support by BacterioScan Inc. The other authors declare that the research was conducted in the absence of any commercial or financial relationships that could be construed as a potential conflict of interest.
