# Supplementary material for: Rapid Phenotypic Detection of Microbial Resistance in Gram-Positive Bacteria by a Real-Time Laser Scattering Method
Source: Front Microbiol. 2017 Jun 14;8:1064. doi: 10.3389/fmicb.2017.01064 (PMC5470558; doi:10.3389/fmicb.2017.01064)
Supplement: Supplementary file 1 [file Presentation_1.pdf]

Supplementary Material

Rapid phenotypic detection of microbial resistance in Gram-positive bacteria by a real-time laser scattering method

Evgeny A. Idelevich, Matthias Hoy, Dennis Görlich, Dennis Knaack, Barbara Grünastel, Georg Peters, Matthias Borowski[#], Karsten Becker[#]*

[#] contributed equally

*** Correspondence:** kbecker@uni-muenster.de

Supplementary Description

Estimating the concentration slope by the SCARM

The figure shows that the concentrations of resistant and susceptible bacteria differed systematically after approximately three or four hours of incubation.

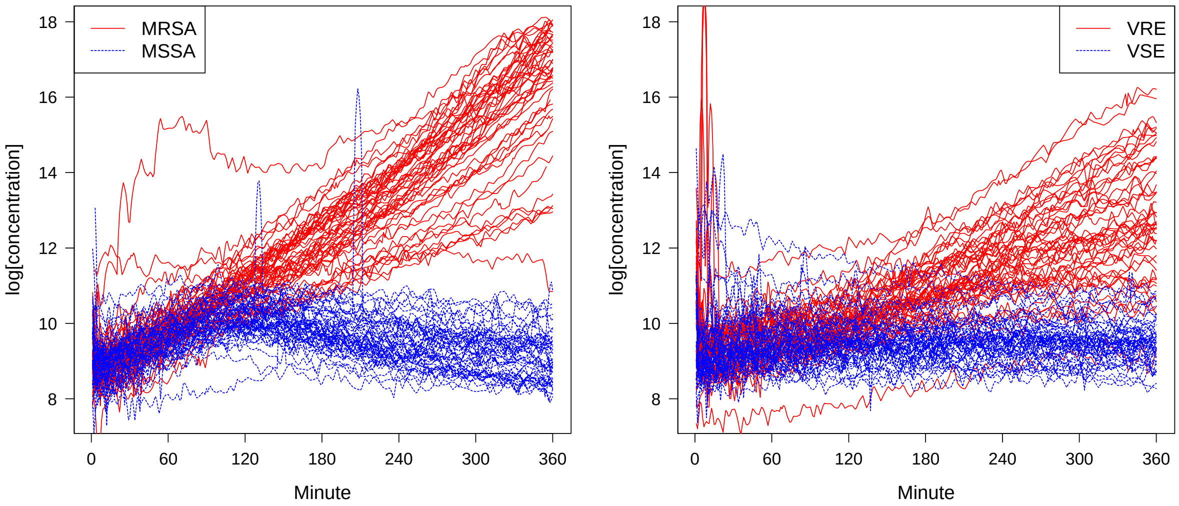

Figure. Concentration measurements of MRSA/MSSA and VRE/VSE (50 samples each) with added antibiotics (no growth controls shown here)

However, the *slopes* (i.e., the gradient or growth per time unit) of the concentration of resistant and susceptible bacteria differed considerably much earlier. Therefore, discrimination between resistant and susceptible bacteria by considering the concentration slopes appears even more promising when time is crucial.

To make this approach feasible in practice, a method is required that computes the current slope in a sequence of concentration measurements in real time; i.e. a method that estimates the current slope as soon as a new measurement is given. This task can be performed by the SCARM (Slope Comparing Adaptive Repeated Median), a method that was developed primarily for smoothing data streams $(x_t)_{t=1,2,...}$ in real time (Borowski and Fried, 2014). As soon as the data stream yields a new observation x_t, the SCARM fits a Repeated Median (Siegel, 1982) regression line to the recent n observations $x_{t-n+1}, \dots, x_t$. The slope of this

regression line can be taken as an estimate of the slope s_t of the data stream at the current time t. The advantage of SCARM is that the number of observations n is not fixed but is adapted to the current data situation at each time t by means of a goodness-of-fit test. This provides a proper regression fit and, thus, a reliable estimate of the current slope of the time series, which is crucial for the feasibility of our approach.

References

Borowski, M., and Fried, R. (2014). Online signal extraction by robust regression in moving windows with data-adaptive width selection. *Statistics and Computing* 24:597-613.

Siegel (1982). Robust regression using repeated medians. *Biometrika* 69:242-244.

Supplementary Table 1. The effect of starting inoculum size on time to detection of five-fold and ten-fold increase of bacterial concentration

Starting inoculum, cfu/ml	*S. aureus*		*E. coli*	
	Time[a] to five-fold increase of bacterial concentration, minutes[b]	Time[a] to ten-fold increase of bacterial concentration, minutes[c]	Time[a] to five-fold increase of bacterial concentration, minutes[d]	Time[a] to ten-fold increase of bacterial concentration, minutes[e]
$1x10^8$	110	127	95	120
$1x10^7$	107	133	98	120
$1x10^6$	121	150	96	129
$5x10^5$	104	159	102	129
$1x10^5$	154	177	156	179
$1x10^4$	204	230	196	216
$1x10^3$	318	341	308	326
$1x10^2$	408	428	381	394
$1x10^1$	502	521	420	438
$1x10^0$	-[f]	-[f]	504	520

[a] Mean from three experiments

[b] ANOVA with Tukey's test for time to detection: no statistically significant difference within the group of higher starting inocula: $1x10^8$, $1x10^7$, $1x10^6$ and $5x10^5$; statistically significant difference between each of these inocula compared to each of other (lower) inocula outside this group; statistically significant difference between each of inocula $1x10^5$, $1x10^4$, $1x10^3$, $1x10^2$ and $1x10^1$ to each of other inocula, except the comparison of $1x10^5$ and $1x10^6$ and $1x10^8$ to each other (no statistically significant difference).

[c] ANOVA with Tukey's test for time to detection: no statistically significant difference within the group of higher starting inocula: $1x10^8$, $1x10^7$, $1x10^6$ and $5x10^5$; statistically significant difference between each of these inocula compared to each of other (lower) inocula outside this group; statistically significant difference between each of inocula $1x10^5$, $1x10^4$, $1x10^3$, $1x10^2$ and $1x10^1$ to each of other inocula, except the comparison of $1x10^5$, $5x10^5$ and $1x10^6$ to each other (no statistically significant difference).

[d] ANOVA with Tukey's test for time to detection: no statistically significant difference within the group of higher starting inocula: $1x10^8$, $1x10^7$, $1x10^6$ and $5x10^5$; statistically significant difference between each of these inocula compared to each of other (lower) inocula outside this group; statistically significant difference between each of inocula $1x10^5$, $1x10^4$, $1x10^3$, $1x10^2$, $1x10^1$ and $1x10^0$ to each of other inocula, except the comparison of $1x10^2$ and $1x10^1$ to each other (no statistically significant difference) and the comparison of $1x10^5$ and $1x10^4$ to each other (no statistically significant difference).

[e] ANOVA with Tukey's test for time to detection: no statistically significant difference within the group of higher starting inocula: $1x10^8$, $1x10^7$, $1x10^6$ and $5x10^5$; statistically significant difference between each of these inocula compared to each of other (lower) inocula outside this group; statistically significant difference between each of inocula $1x10^5$, $1x10^4$, $1x10^3$, $1x10^2$, $1x10^1$ and $1x10^0$ to each of other inocula, except the comparison of $1x10^2$ and $1x10^1$ to each other (no statistically significant difference) and the comparison of $1x10^5$ and $1x10^4$ to each other (no statistically significant difference).

[f] The starting inoculum of $1x10^0$ cfu/ml for *S. aureus* neither grew in the instrument nor resulted in colonies on vital count plates in two of three experiments, and was therefore excluded from the analysis.

Supplementary Table 2. The effect of different liquid media on time to detection of five-fold and ten-fold increase of bacterial concentration

Broth	*S. aureus*		*E. coli*	
	Time[a] to five-fold increase of bacterial concentration, minutes	Time[a] to ten-fold increase of bacterial concentration, minutes	Time[a] to five-fold increase of bacterial concentration, minutes	Time[a] to ten-fold increase of bacterial concentration, minutes
CA-MHB	133	187	99	126
BHI	104	159	102	129
TSB	151	306	108	130
LB	111	146	111	146

CA-MHB, cation-adjusted Mueller-Hinton broth; BHI, brain-heart infusion broth; TSB, tryptic soy broth; LB, lysogeny broth

[a] Mean from three experiments

No statistically significant difference between different broths

Supplementary Table 3. The effect of broth filtration on time to detection of five-fold and ten-fold increase of bacterial concentration

Broth	*S. aureus*				*E. coli*			
	Time[a] to five-fold increase of bacterial concentration, minutes		Time[a] to ten-fold increase of bacterial concentration, minutes		Time[a] to five-fold increase of bacterial concentration, minutes		Time[a] to ten-fold increase of bacterial concentration, minutes	
	CA-MHB	BHI	CA-MHB	BHI	CA-MHB	BHI	CA-MHB	BHI
Filtered	167	127	207	174	128	142	155	189
Unfiltered	142	97	235	148	144	118	172	144

CA-MHB, cation-adjusted Mueller-Hinton broth; BHI, brain-heart infusion broth

[a] Mean from three experiments

No statistically significant difference between different broths

The cut-off in increase of bacterial concentration was not achieved in one of three experiments with CA-MHB for both *S. aureus* and *E. coli*. The mean presented is the mean from two experiments

Supplementary Table 4. Accuracy of the approaches (a)-(d) to discriminate between methicillin-resistant and methicillin-susceptible *S. aureus*; sensitivities (SE) and specificities (SP) were estimated by leave-one-out cross-validation

Approach	(a)				(b)				(c)				(d)			
**Min.**	**SE (%)**	**(95% CI)**	**SP (%)**	**(95% CI)**	**SE (%)**	**(95% CI)**	**SP (%)**	**(95% CI)**	**SE (%)**	**(95% CI)**	**SP (%)**	**(95% CI)**	**SE (%)**	**(95% CI)**	**SP (%)**	**(95% CI)**
60	84	(70.9-92.8)	46	(31.8-60.7)	52	(37.4-66.3)	70	(55.4-82.1)	64	(49.2-77.1)	62	(47.2-75.3)	46	(31.8-60.7)	76	(61.8-86.9)
120	80	(66.3-90)	80	(66.3-90)	78	(64-88.5)	84	(70.9-92.8)	78	(64-88.5)	84	(70.9-92.8)	66	(51.2-78.8)	90	(78.2-96.7)
180	94	(83.5-98.7)	94	(83.5-98.7)	86	(73.3-94.2)	98	(89.4-99.9)	98	(89.4-99.9)	96	(86.3-99.5)	84	(70.9-92.8)	98	(89.4-99.9)
240	100	(92.9-100)	100	(92.9-100)	92	(80.8-97.8)	94	(83.5-98.7)	100	(92.9-100)	100	(92.9-100)	100	(92.9-100)	100	(92.9-100)
300	100	(92.9-100)	100	(92.9-100)	98	(89.4-99.9)	98	(89.4-99.9)	98	(89.4-99.9)	100	(92.9-100)	98	(89.4-99.9)	100	(92.9-100)
360	97.6	(87.4-99.9)	98	(89.4-99.9)	97.6	(87.4-99.9)	98	(89.4-99.9)	97.6	(87.4-99.9)	100	(89.7-100)	97.6	(87.4-99.9)	100	(89.7-100)

SE: Sensitivity, SP: Specificity; (a) Discrimination based on concentrations, (b) Discrimination based on ratios of concentrations (sample with antibiotics vs. growth control), (c) Discrimination based on concentration slopes, (d) Discrimination based on ratios of concentration slopes (sample with antibiotics vs. growth control)

Supplementary Table 5. Accuracy of the approaches (a)-(d) to discriminate between vancomycin-resistant and vancomycin-susceptible *E. faecium*; sensitivities (SE) and specificities (SP) were estimated by leave-one-out cross-validation

Approach	(a)				(b)				(c)				(d)			
**Min.**	**SE (%)**	**(95% CI)**	**SP (%)**	**(95% CI)**	**SE (%)**	**(95% CI)**	**SP (%)**	**(95% CI)**	**SE (%)**	**(95% CI)**	**SP (%)**	**(95% CI)**	**SE (%)**	**(95% CI)**	**SP (%)**	**(95% CI)**
60	88	(75.7-95.5)	30	(17.9-44.6)	90	(78.2-96.7)	42	(28.2-56.8)	56	(41.3-70)	48	(33.7-62.6)	32	(19.5-46.7)	78	(64-88.5)
120	72	(57.5-83.8)	66	(51.2-78.8)	78	(64-88.5)	82	(68.6-91.4)	86	(73.3-94.2)	70	(55.4-82.1)	74	(59.7-85.4)	82	(68.6-91.4)
180	90	(78.2-96.7)	84	(70.9-92.8)	88	(75.7-95.5)	82	(68.6-91.4)	96	(86.3-99.5)	90	(78.2-96.7)	94	(83.5-98.7)	98	(89.4-99.9)
240	86	(73.3-94.2)	90	(78.2-96.7)	92	(80.8-97.8)	78	(64-88.5)	90	(78.2-96.7)	92	(80.8-97.8)	96	(86.3-99.5)	94	(83.5-98.7)
300	88	(75.7-95.5)	98	(89.4-99.9)	94	(83.5-98.7)	74	(59.7-85.4)	98	(89.4-99.9)	96	(86.3-99.5)	98	(89.4-99.9)	92	(80.8-97.8)
360	90	(78.2-96.7)	100	(92.9-100)	96	(86.3-99.5)	76	(61.8-86.9)	96	(86.3-99.5)	96	(86.3-99.5)	96	(86.3-99.5)	86	(73.3-94.2)

SE: Sensitivity, SP: Specificity; (a) Discrimination based on concentrations, (b) Discrimination based on ratios of concentrations (sample with antibiotics vs. growth control), (c) Discrimination based on concentration slopes, (d) Discrimination based on ratios of concentration slopes (sample with antibiotics vs. growth control)

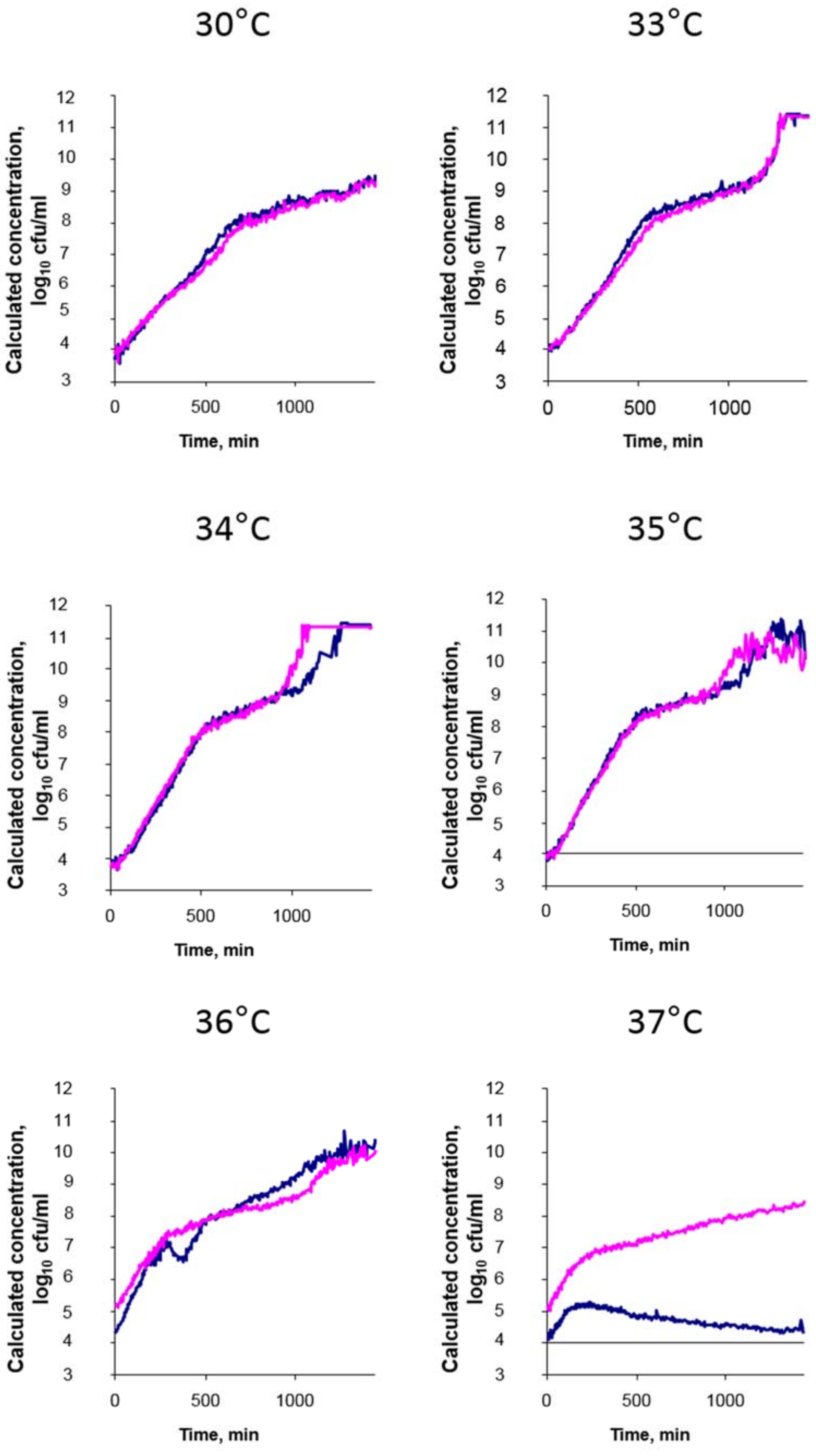

Supplementary Figure 1. Effect of incubation temperature on phenotypic detection of methicillin resistance in *S. aureus* using real-time laser-scattering method. The samples with

antibiotic are indicated in blue, and the growth control samples without antibiotic are indicated in red. The growth of the MRSA reference strain ATCC BAA-44 was, as expected, not inhibited by cefoxitin in breakpoint concentration 4 μg/ml at incubation temperatures 30°C, 33°C, 34°C, 35°C, 36°C, but at the temperature 37°C complete inhibition was observed.

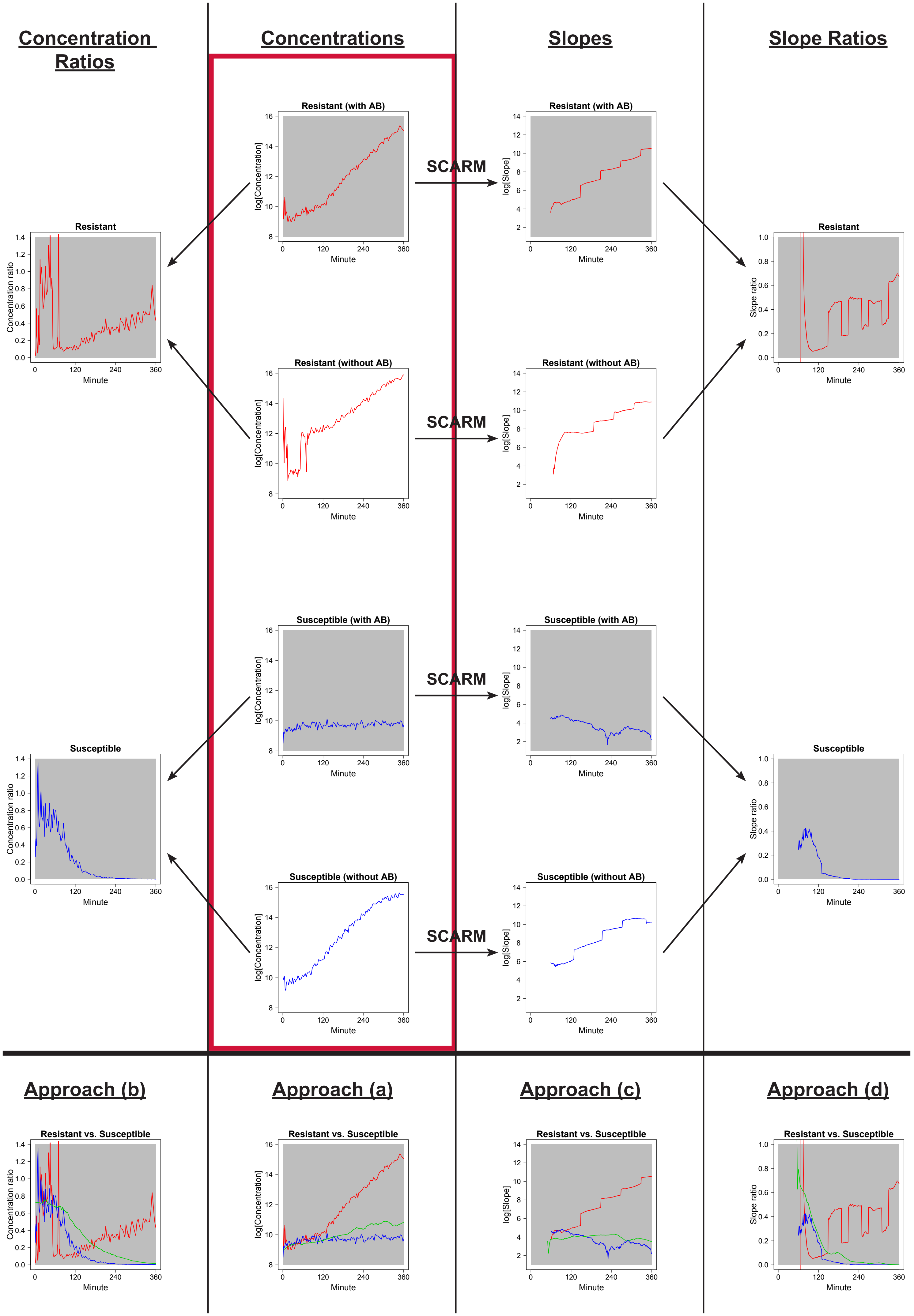

Concentration Ratios
Concentrations
Slopes
Slope Ratios
Resistant (with AB)
Resistant (without AB)
Susceptible (with AB)
Susceptible (without AB)
Resistant
Susceptible
SCARM
SCARM
SCARM
SCARM
log[Concentration]
log[Slope]
Concentration ratio
Slope ratio
Minute
Approach (b)
Approach (a)
Approach (c)
Approach (d)
Resistant vs. Susceptible
Resistant vs. Susceptible
Resistant vs. Susceptible
Resistant vs. Susceptible

Supplementary Figure 2. Laser scattering delivered time series of concentration measurements of resistant and susceptible isolates with and without antibiotics (AB), marked by the red colored box. (For simplicity, only one out of the n=50 time series is shown here in each plot.) **Approach (a):** if the concentration measurements *with* AB are greater than certain cutoffs (calculated by ROC analysis, green time series), we propose to decide for resistance and otherwise for susceptibility. This approach does not require additional growth controls, i.e. additional measurements of concentration *without* AB. **Approach (b):** if the concentration *ratios* are greater than certain cutoffs (calculated by ROC analysis, green time series), we propose to decide for resistance and otherwise for susceptibility. The concentration ratio at time t is the concentration of an isolate with AB divided by the concentration of its growth control, each at time t. **Approach (c):** if the concentration *slopes* are greater than certain cutoffs (calculated by ROC analysis, green time series), we propose to decide for resistance and otherwise for susceptibility. We use the SCARM to estimate the slopes in real-time. **Approach (d):** if the *slope ratios* are greater than certain cutoffs (calculated by ROC analysis, green time series), we propose to decide for resistance and otherwise for susceptibility. The slope ratio at time t is the slope of an isolate with AB divided by the slope of its growth control, each at time t. We use the SCARM to estimate the slopes in real-time.
